# Unusual, stable replicating viruses generated from mumps virus cDNA clones

**DOI:** 10.1371/journal.pone.0219168

**Published:** 2019-07-05

**Authors:** Connor Bamford, Elizabeth Wignall-Fleming, Vattipally B. Sreenu, Richard Randall, Paul Duprex, Bertus Rima

**Affiliations:** 1 Centre for Virus Research, Glasgow University, Glasgow, Scotland, United Kingdom; 2 Centre for Experimental Medicine, Queen’s University Belfast, Belfast Northern Ireland, United Kingdom; 3 School of Biology, St Andrews University, St Andrews, Scotland, United Kingdom; 4 Center for Vaccine Research, University of Pittsburgh, Pittsburgh, Pennsylvania, United States of America; Universitat Bern, SWITZERLAND

## Abstract

In reverse genetic experiments we have isolated recombinant mumps viruses (rMuV) that carry large numbers of mutations clustered in small parts of their genome, which are not caused by biased hyper-mutation. In two separate experiments we obtained such recombinant viruses: one virus had 11 mutations in the V/P region of the genome; the other, which also contained an extra transcription unit encoding green fluorescent protein (EGFP), had 32 mutations in the N gene. These specific sets of mutations have not been observed in naturally occurring MuV isolates. Unusually, the vast majority of the mutations (48/51) were synonymous. On passage in Vero cells and human B-LCL cells, a B lymphocyte-like cell line, these mutations appear stable as no reversion occurred to the original consensus sequence, although mutations in other parts of the genome occurred and changed in frequency during passage. Defective interfering RNAs accumulate in passage in Vero cells but not in B-LCL cells. Interestingly, in all passaged samples the level of variation in the EGFP gene is the same as in the viral genes, though it is unlikely that this gene is under any functionality constraint. What mechanism gave rise to these viruses with clustered mutations and their stability remains an open question, which is likely of interest to a wider field than mumps reverse genetics.

## Introduction

Mumps virus (MuV) is a human pathogenic RNA virus in the genus *Rubulavirus* in the family *Paramyxoviridae* [1]. This family of non-segmented negative stranded RNA viruses shares basic replication strategies with the other viruses in the order *Mononegavirales*. MuV has a genome of 15,384 nucleotides (nt) in length, which contains 7 transcription units from the 3’ end of the negative stranded genome to the 5’ end respectively. These encode respectively the nucleocapsid protein (N); the innate immune modulatory protein V, the matrix protein (M), the fusion protein (F), a small hydrophobic protein (SH), a haemagglutinin-neuraminidase protein (HN) and the large protein (L) which carries the RNA-dependent RNA polymerase activity (RdRp). The major co-factor for the RdRp required during transcription is the phosphoprotein (P) which is generated by co-transcriptional editing during the copying of the V gene to generate P protein by the insertion of 2 (or 5) non-templated G residues into the nascent transcript. A so-called I protein is also generated by insertion of 1 or 4 G residues. V and I are probably non-structural proteins due to their low abundance in semi purified virus preparations [1]. In the *Rubulavirus* genus there is no overlapping open reading frame (ORF) for the C protein in the gene encoding the V and P proteins and unedited mRNAs transcribed from the MuV genome encode the V protein and not the P protein as is the case in many of the other *Paramyxoviridae* [2,3]. No overlapping ORFs have been identified in the N gene of MuV.

Reverse genetics of MuV and other members of the order *Mononegavirales* from plasmids that encode the entire genome of the virus has been described more than two decades ago [4] and this system has been used to elucidate aspects of mumps virus pathogenesis and virulence in clinical isolates [4–8]. In this, it is not different from other viruses in the order *Mononegavirales*. Recently, we established a ‘rescue’ system based on the sequence of MuV in clinical tissue so that we could study the properties and behaviour of viruses that had not been passaged in cultured cells and thereby potentially exposed to selective pressures exerted by the host cell *in vitro* [9]). Recombinant viruses were rescued on Vero cells (MuV^G09^) from the clinical material and proved to be genotype G virus, similar to other viruses isolated in the US during recent outbreaks [10].

MuV is stable in the field and substitution rates have been estimated to be approximately 9.1 x 10^−4^ sub/site/y per annum [11]. No direct experimentally determined mutation rates have been reported for MuV. Here, we report that we obtained a number of rescued viruses that carried unusual clusters of mutations in the N and V/P genes, which involved stable synonymous nucleotide changes that have not been observed in natural isolates in any of the genotypes of MuV hitherto described.

## Materials and methods

### Viruses and cells

Clinical material was obtained from Dr Paul Rota (CDC Atlanta) as a buccal swab from a patient during the 2009/10 US outbreak of mumps in New York caused by a genotype G5 virus [9, 10]. The standardised name for the isolate is: MuV/New York.USA/2009 [G]. We isolated the virus on Vero cells, passaged it four times in Vero cells and called it MuV^G09^. The complete consensus sequences of the viral RNA in the clinical sample and the isolated viruses were determined by Sanger sequencing of overlapping RT-PCR amplicons (sequences of primers available on request) and found to be identical [9].

Viral RNA was isolated by phenol/chloroform extraction of semi purified virus preparation prepared by pelleting the virus through a 25% sucrose cushion by ultracentrifugation using TRIzol solution or from virus-containing supernatant using TRIzol LS solution (Thermo Fisher Scientific). The cDNA was produced using SuperScript III (SSIII) kit followed by PCR by Taq DNA polymerase to generate overlapping amplicons covering the entire genome. Amplicons were purified using the QIAquick PCR Purification Kit (Qiagen), resuspended in TE buffer before sequencing using BigDye Terminator v3.1 sequencing kit. Full-length consensus sequences were generated and chromatograms were inspected manually to identify mixed sequences.

Vero cells were used for routine passage of the viruses as well as titration of plaque forming units (pfu). Six well trays seeded with confluent monolayers of Vero cells were infected with various dilutions of supernatant virus stocks and stained 2 days after infection with methylene-blue to detect and count plaques. B-LCL cells, an EBV transformed primary B cell line, were obtained from Erasmus University Medical Centre, Rotterdam [12].

### Generation of recombinant viruses

Plasmids that expressed the N, P and L proteins with the authentic sequence of the MuV^G09^ virus were generated to act as helper plasmids in the rescue experiments. Plasmids were also generated that represented the full length consensus sequence of the viral RNA which was identical to that of the isolated virus MuV^G09^ as well as one in which the enhanced green fluorescent protein gene (EGFP) was inserted as an additional transcription unit between the V/P gene and the M gene of MuV (referred to as position 3). The complete antigenomic MuV sequence was placed between a T7 promotor and hepatitis delta ribozyme by sequential cloning of cDNA fragments using endogenous restriction enzyme sites. Introduction of the EGFP transcription was carried out using endogenous restriction enzyme sites and the open reading frame was flanked by M 5’ UTR and V/P 3’ UTR. The sequence of the full-length antigenomic plasmids was identical to the consensus sequence of the clinical material. Vero cells (90% confluency in a 6-well dish) were infected with Fowl Pox virus (FP) that expressed T7 RNA polymerase [13] (MOI = 0.5 TCID50) by spin-inoculation (30 mins at 300g). At 1 h.p.i. the FP T7 inoculum was removed and a four-plasmid mix was added to the cells with Lipofectamine 2000 reagent: pCGMuV^G09^N (1 μg), pCG-MuVG09P (0.6 μg) and pCG-MuVG09L (0.4 μg) and pMuVG09 (5 μg). The quantities of each of the plasmids used are similar to those used by us in other reverse genetics experiments of viruses in the *Paramyxoviridae* family. Opti-MEM (2 ml) was added and cells were incubated for 18 hours at 37°C with 5% (v/v) CO2. The growth medium was changed to DMEM containing 5% (v/v) foetal calf serum and the appearance of syncytia and/or EGFP-expressing cells was monitored over 1 week. Usually never more than one syncytium was observed per well. These were aspirated and propagated further on Vero cells for 4 low MOI passages before sequencing, passaging the virus when maximum CPE had been observed.

### Next generation sequencing (NGS)

The RNA was extracted from infected cells using Trizol and subjected to directional library preparation using the TruSeq Stranded mRNA Library Prep Kit (Illumina) omitting polyA selection using oligo–dT beads as per manufacturer’s instructions. The samples were sequenced using the Mi-seq illumina platform generating paired end reads of ~ 150 nucleotides in length. The reads were trimmed to remove adaptor sequences and any reads with low quality scores were removed from the analysis. The sequencing data were subjected to directional analysis [14] which separates the reads based on directionality allowing the isolation of the viral genome (negative sense) and viral antigenome reads (positive sense). The isolated viral genome reads were then aligned to the MuV reference sequence using BWA alignment software version 0.7.5a-r405. Variants were assessed visually for their positions in reads using Tablet [15]. An in-house script was then used to enumerate the SNPs at each nucleotide of the reference sequence (available from Dr Vattipally Sreenu, University of Glasgow). The full NGS data set is available at study number PRJNA545057.

## Results

### Generation of recombinant MuV with clusters of mutations

Our attempts to obtain rMuV virus failed in all cases to generate a virus with the same sequence to that in the plasmid after aspirating the material of a primary syncytium from a single well and passaging the inoculum 4 times on Vero cells at low MOI. The reverse genetics system used ‘helper’ expression plasmids representing the authentic N, P and L genes of MuV^G09^ and a plasmid containing the consensus sequence of MuV^G09^ so that fowl pox virus mediated recombination between helper plasmids and the full length genome plasmid could not lead to potential sequence alterations. Out of the 5 plaque picks that were made in the primary rescue wells transfected with a plasmid representing the full length genome of MuV^G09^ none were identical to the input cDNA clone sequence and a combined total of 18 mutations were observed. Four retrieved viruses had one or two mutations but one virus rMuV^G09^PP1 (abbreviated as PP1) had 11 mutations in the V/P gene (Table 1) between nucleotides 2551 and 2867 spacing mutations on the average 32 nt from each other. This area encodes the C terminal end of the V protein and part of the more conserved C-terminal domain of the P protein in MuV. Nine mutations were synonymous and two of the PP1 mutations led to amino acid changes at position 192 (P>L) and 212 (Q>P) in the P protein. Both mutations were synonymous in the overlapping V protein reading frame.

**Table 1:** Stability of the unique mutations in RNA extracted from semi-purified MuV–PP1 virus after 6 passages on Vero cells.

Position nr in the genome	Change from G09 to PP1	Effect in PP1 on protein, position and change	Number of reads in PP1 passage 6	Variant reads	Number of reads in MuV^G09^ passage 6	Variant reads
2551	C>U	P192 P>L; V191 P syn	276 U	none	1606 C	1 A
2611	A>C	P212 Q>P V211 P syn	207 C	**1 A**; 2 U	1171 A	1 A; 1 C
2654	A>G	P226 R syn	206 G	**3 A**	986 A	none
2763	C>U	P263 L syn	222 U	none	1544 C	1 U
2768	U>A	P264 A syn	232 A	none	1312 U	3 C; 1 G
2780	A>G	P268 G syn	206 G	none	1417 A	none
2789	G>A	P271 A syn	265 A	**1 G**	1635 G	1 U
2810	G>U	P278 P syn	261 U	none	2149 G	2 U
2816	C>U	P280 N syn	266 U	1 A; 2 G	1645 C	2 A
2864	U>C	P296 H syn	280 C	none	1714 U	none
2867	A>G	P297 V syn	294 G	none	1609 A	none
Total			2715	10	16788	frequency 0.8 x 10^−3^

Syn = synonymous; in bold variants that would restore the original nucleotide in MuV^G09^

Rescue was also attempted from a plasmid into which the EGFP gene had been inserted between the V/P and M genes of mumps virus MuV^G09^. Similarly, out of the 7 plaque picked viruses none had a nucleotide sequence identical to the original plasmid. Six out of the 7 viruses carried *in toto* 9 mutations (8 non-synonymous replacements and one A insertion in the poly-adenylation signal in the F gene). However, one plaque picked virus rMuV^G09^EGFP(3)PP2 virus (abbreviated here as PP2) had 32 mutations in two clusters in the N gene (Table 2). One cluster contained 13 mutations between nt 607 and 860 distancing mutations by on average 21 nucleotides. This area encodes a relatively conserved part of the N protein of MuV. The second cluster contained 19 mutations between nucleotides 1225 and 1558 with an average spacing of 18 nucleotides. This encodes part of the relatively variable C terminal tail of the N protein of MuV. In PP2 almost all the mutations (31/32) were synonymous with the exception of a single conservative V>A mutation at position 460 of the N protein. None of the mutations observed in PP1 and PP2 were observable as minor peaks in sequencing chromatograms of the sequencing reactions carried out on the clinical material. The sets of mostly synonymous mutations observed in these viruses in the N and V/P genes were also not present as linked variations in sequence of the mumps virus genotypes in the databanks. No clusters of mutations were observed in the other genes of MuV by Sanger sequencing of the primary isolates.

**Table 2:** Stability of the unique mutations in RNA extracted from semi-purified PP2 virus after 6 passages on B-LCL cells.

Position nr in the genome	Change from G09 to PP2	antigenome reads in PP2 passage 6	Variant reads	genome reads in PP2 passage 6	Variant reads
607	G > A syn	846 A	1 C	850 A	2 C
622	A > G syn	1031 G	none	1001 G	none
628	A > G syn	1067 G	4 U	1059 G	none
679	C > A syn	680 A	none	1506 A	1 G; 1 U; **1 C**
697	U > G syn	692 G	none	2502 G	**3 U**
736	G > U syn	887 U	5 C; **2 G**	2733 U	6 A; 1 C; **7 G**
760	G > U syn	1093 U	4 C; **3 G**	1594 U	**1 G**
784	A > G syn	975 G	**3 A**	1348 G	6 U; **2 A**
841	U > C syn	1338 C	**2 U**; 2 A; 2 G	659 C	2 A
844	U > C syn	1400 C	3 A; **3 U**	650 C	2 A; 1 G
850	U > C syn	1447 C	**1 U**; 1 G	632 C	1 G; 1 **U**
859	A > G syn	1444 G	2 U	523 G	none
860	C > U syn	1434 U	**3 C;** 3 G	489 U	none
Total frequency	cluster 1	14334	43	15546	37 1.04 x 10^−3^
1225	C > U syn	1325 U	**2 C**	620 U	**1 C**; 1 G
1240	G > A syn	1461 A	6 C; 1 U	514 A	1 C
1312	A > G syn	2819 G	2 U; **1 A**	369 G	2 U
1327	C > U syn	2613 U	**6 C**; 2 G; 1 A	339 U	**1 C**
1330	U > C syn	2645 C	10 A; 8 U; 1 G	343 C	**1 U**
1354	A > G syn	2759 G	**12 A**; 2 C; 4 U	488 G	none
1366	G > A syn	2811 A	**5 G**; 2 C; 1 U	764 A	**3 G**; 1U
1369	A > G syn	2836 G	9 U; **3 A**; 2 C	784 G	1 C; 1 U
1384	G > C syn	2596 C	6 A; 3 U	1195 C	3 A; 1 U
1387	G > A syn	2610 A	8 C; 2 U; **1 G**	1322 A	1 U1]
1390	C > U syn	2584 U	4 A; 2 G	1429 U	3 A; 3 G
1413	G > A syn	3626 A	3 C; 2 U; **1 G**	1612 A	3 U; 1 C
1438	G > A syn	3749 A	6 C; **5 G**; 3 U	1553 A	8 C; 3 U; **1 G**
1451	U > C syn	3508 C	5 A; **3 U**; 2 G	1510 C	3 A; **1 U**
1468	C > U syn	4457 U	**5 C**; 3 G; 2 A	982 U	**4 C**; 2 G; 1 A
1471	U > C syn	4496 C	3 A; **2 U**	1167 C	2A; **1 U**
1489	U > C syn	4259 C	**7 U**; 5 A	1352 C	**2 U**
1524	U > C N460 V>A	4359 C	5 A; **3 U**	1265 C	4 A; 1 G; **1 U**
1558	G > A syn	2910 A	3 U; 2 C; **1 G**	729 A	**1 G**; 1 U; 1 C
Total frequency	cluster 2	58423	176	18337	65 1.05 x 10^−3^
Total	both clusters	72757	To original: 81 To other: 138 Other/original = 1.70	33883	To original: 31 To other: 71 other/original > 2.29

In bold variants that would restore the original nucleotide in MuV^G09^

**Fig 1:**
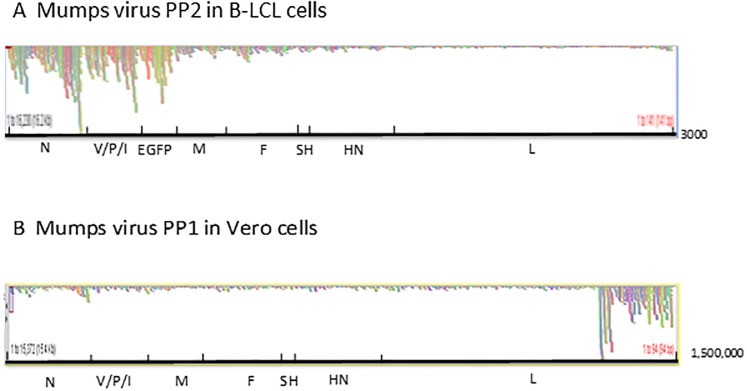
Coverage of reads and preponderance of defective interfering RNAs in passages of viruses on Vero and B-LCL cells. Coverage of reads and preponderance of Defective Interfering RNAs after 6 passages of viruses on Vero and B-LCL cells.

The original mutations in the V/P gene of PP1 and the N gene of PP2 first determined by Sanger sequencing of the semi purified virus stocks were confirmed in the Next Generation Sequencing (NGS) studies described below, with the exception of a deletion mutation in the L gene in the recombinant PP1 virus. In PP1, Sanger sequencing of the original plaque picked virus identified one extra deletion mutation in the L gene (15127–138), which would have led to a premature termination of the L protein. However, in NGS, the non-deleted sequence appeared to be present as a minor species (<5%) and not detectable in the original chromatograms obtained for the Sanger sequencing. Thus, it is likely that a deletion in defective interfering (DI) particles masked the standard virus sequence in Sanger sequencing because of their preponderance. The absence of a type I interferon production in African Green Monkey kidney Vero cells allows the accumulation of defective interfering (DI) particles in MuV passages [16], which give rise to highly fluctuating titres in the passages (S1 Table). In contrast, viruses populations passaged on B-LCL cells did not appear to contain DI particles, as the high coverage at the 3’ end of the antigenomic sequence (which represents the L gene and the region covered by DI particles) present in Vero cell passaged virus is absent in virus passaged on B-LCL cells (Fig 1). The accumulation of DI particles in virus passaged on Vero cells is also indicated by the substantial number of variant reads observed at the 3’end of the antigenome in virus populations passaged on Vero cells (S2 and S3 Tables) and furthermore by the observation that in plaque assays the lowest dilution with the highest number of pfu did not show any plaques (S1 Table) as at high concentrations DI particles prevent plaque formation.

Diagrams that show the total numbers of reads obtained in NGS for each nucleotide over the entire mumps genome of 15372 nucleotides (x-axis) displayed by the Tablet programme [15]. In Fig 1A, the y-axis scale is 1–3000 in Fig 1B it is 1–1,500,000 indicating the accumulation of reads associated with the appearance of DI particles at the 3’ end of the antigenome.

**Fig 2:**
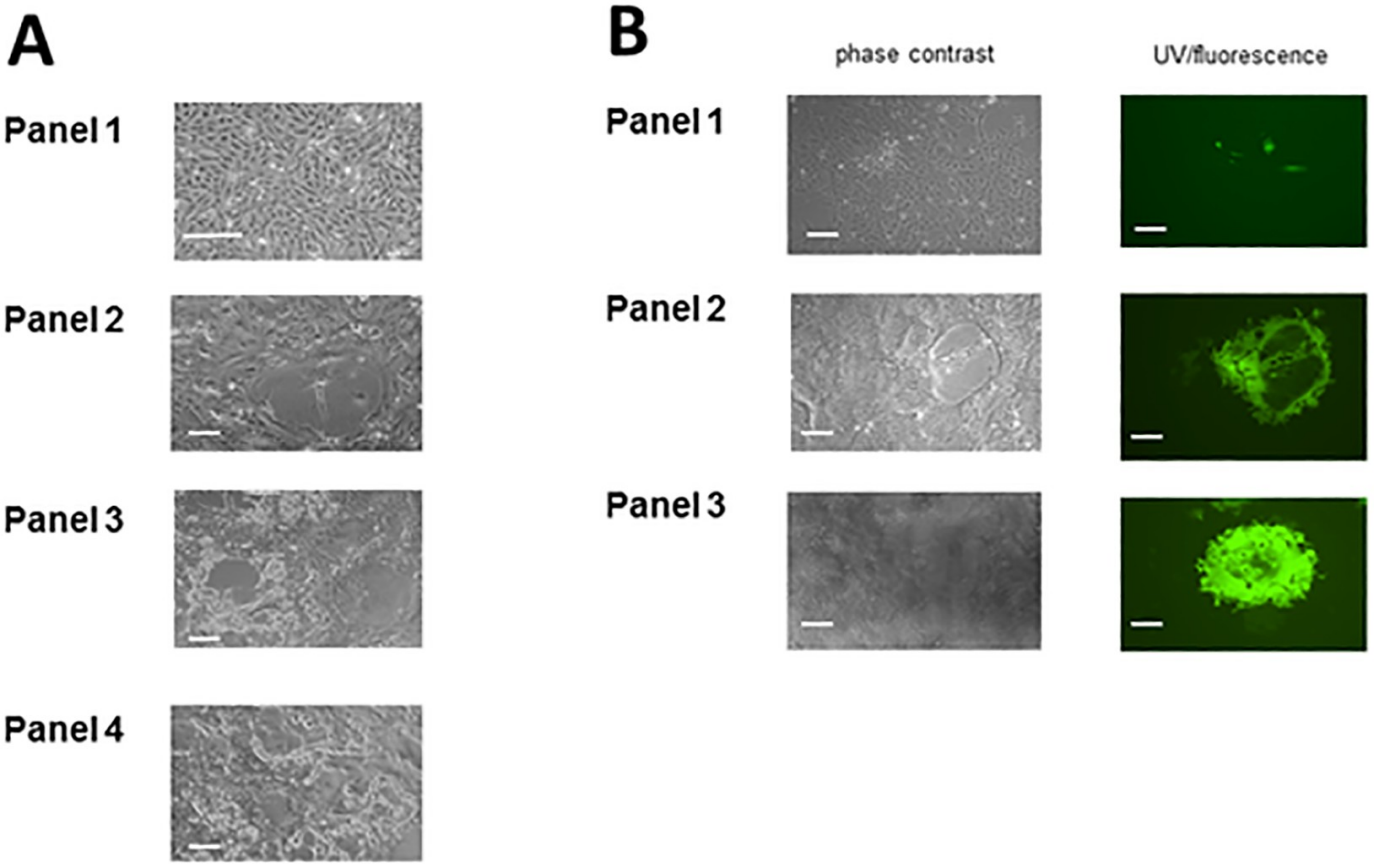
Rescue of the viruses as replicators; cpe and fluorescent plaques. A: Generation of rMuV^G09^ by reverse genetics. Panel 1 shows mock infected Vero cells; panel 2 shows the presence of primary foci of rescue at 5 days post transfection; panel 3 shows primary syncytia which were plaque picked and subsequently Vero cells were infected with the aspirated virus stocks. CPE was detected 1–2 dpi. Panel 4 shows plaque picked rMuV^G09^ grown for 4 low MOI passages on Vero cells. All show characteristic syncytium-formation (scale bar is 50μ). B: Generation of rMuV^G09^ expressing EGFP—rMuV^G09^EGFP(3)—by reverse genetics. Panel 1 shows the presence of primary foci of rescue at 5 days post transfection in both phase contrast and UV microscopy; panel 2 shows primary syncytia which were plaque picked and subsequently Vero cells were infected with the aspirated virus stocks. EGFP expression was evident 1 dpi and cpe was detected 1–2 dpi. Panel 3 shows plaque picked rMuV^G09^EGFP(3) grown for 4 low MOI passages on Vero cells. Passaged virus images show characteristic syncytium formation (scale bar is 50μ).

Both PP1 and PP2 grew to similar titres and generated the same type and level of cytopathic effect in Vero cells (formation of syncytia) at similar times post infection as the original MuV^G09^ non-recombinant progenitor virus isolated from the clinical material on Vero cells. In the case of PP2 these syncytia showed green fluorescence (Fig 2). The cytopathic effects of PP2 and MuV^G09^ on B-LCL cells were the same (cell death and occasional fusion in clumps of cells) and green fluorescence was observable in the clumps of the PP2 infected B-LCL cells floating in the medium. The growth characteristics of both PP1 and PP2 viruses were similar to those of their progenitor virus and hence we did not carry out experiments to assess their competitive advantage or disadvantage in mixed infection experiments.

### Assessment of the stability of the sets of mutations by NGS

Six additional passages of both viruses were carried out with an undiluted inoculum provide maximum opportunity for the fixation of mutant genomes. PP1 was passaged on Vero cells and PP2 on B-LCL cells. As a control we also passaged the non-recombinant MuV^G09^ virus on Vero and B-LCL cells under the same conditions as PP1 and PP2. We chose these two cell substrates because of their different biological properties. Vero cells are adherent and give rise to syncytia. Vero cell passages of MuV were carried out by infection of fresh Vero cell monolayers with undiluted supernatant virus from the previous passage. In the B-LCL cells, which are an IFN competent human B lymphocyte cell line that grow in suspension and leads to large cell clumps, we choose to allow maximum chances for the accumulation of mutations by carrying out the passages in such way that each passage represents an addition of fresh uninfected cells to the culture medium in which the cell clumps are dispersed by gentle shaking. The supernatant virus of each passage in B-LCL cells was titrated on Vero cells (S1 Table).

The supernatant viruses from the Vero and B-LCL cell passages were pelleted by ultracentrifugation through a 25% (w/v) sucrose cushion and directionally analysed by NGS using a library preparation method that retains directionality of the purified RNA so that the polarity of each read (positive-antigenomic or negative-genomic strand) could be determined on an Illumina platform.

Table 1 demonstrates that the set of the 11 mutations in the V/P gene of PP1 was maintained and stable over the 6 passages. The stability of PP1 in the passage series was not significantly different to that of the non-recombinant MuV^G09^ virus also passaged 6 times in parallel experiments on Vero cells. Similarly, the 32 mutations in PP2 (Table 2) were also stable on passage in B-LCL cells and as stable as the original MuV^G09^ nucleotides at these positions in parallel passages of the MuV^G09^ virus. The frequencies with which the alternative readings occur in each cluster were in the order of 0.001 or 0.1% This in our experience is the normal frequency of alternative reads (0.04 to 0.10%) in NGS sequencing projects which may be generated during the amplifications involved in the library preparation and the sequence reading process itself. No significant predilection for changes that would restore the original MuV^G09^ nucleotide at any given position in the cluster of mutations was observed. The two other possible nucleotides at the mutated position were observed approximately twice as frequent (1.7–2.3) as those that would restore the original MuV^G09^ nucleotide at that position. This is what would be expected if the direction of variation was random rather than directed.

### Evolution of cluster-independent mutations during passage of rMuV

**Fig 3:**
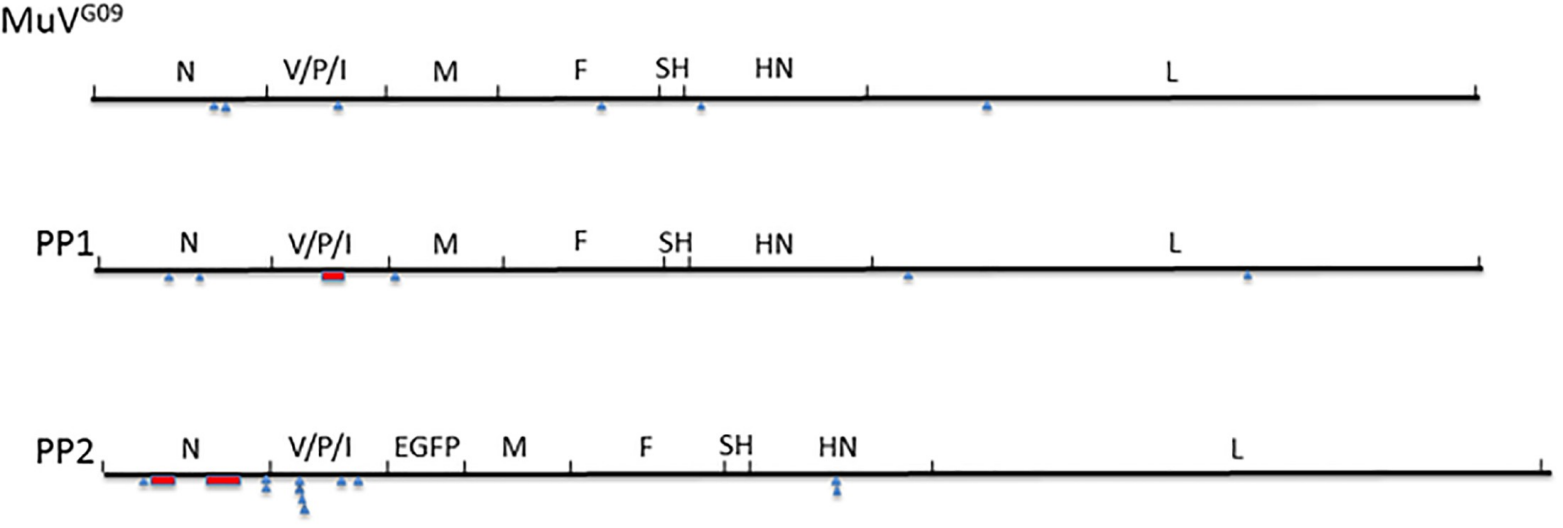
Genome distribution of mutations occurring during passage. The position of new mutants observed after six passages of MuV^G09^ and PP1 on Vero cells and PP2 virus on B-LCL cells. Red boxes indicate the position of the clusters of mutations in the genomes of PP1 and PP2 virus; blue triangles show the genome positions of the mutants identified in Table 3.

In order to ascertain that the passaging conditions did not impose some unexpected artefactual sequence stability we assessed whether mutations occurred during the passage series outside the clusters present in PP1 and PP2. Many specific mutations accumulated to high frequencies during the six passages outside the clusters of originally mutated residues in PP1 and PP2. This indicates that though the virus could mutate in response to the changed cellular environment, the original sets of mutations were stably maintained. Examples of changes occurring during passage are given in Table 3 and their distribution is depicted in Fig 3. The more comprehensive representation of all the changes observed at a frequency of >1% in the deep sequencing reads are compiled and shown in S2 and S3 Tables for PP1 and MuV^G09^ respectively.

**Table 3:** Examples of novel mutations observed after 6 passages of PP1, PP2 and the parent MuV^G09^ virus.

Virus/cell type	position nr	Passage 1	Passage 6	Effect on protein and position	Frequency
PP1/Vero	849	U	U>G	N235 F>C	11.5%
	1178	G	G>A	N345 V>I	24.6%
	3355	A	A>G	M31 E>G	52%
	9014	U	U>C	L193 I>T	9.9%
	12748	C	C>U	L1437 syn	20%
PP2/B-LCL	559	U	U>C	N138 syn	3%
	1896	U	U>C	N3’UTR	7%
	1897	U	U>A	N3’UTR	6.6%
	2710	A	A>G	P245 Q>R	33%
	2859	A	A>G	P295 D>N	7.6%
	8171	G	G>A	HN238 G>S	38%
	8175	U	U>G	HN239 L>R	46%
	2227	U	U>C	P/V83 syn	6%
	2251	U	U>C	P/V91 syn	6%
	2256	U	U>C	P/V93 I>T	6%
	2260	U	U>C	P/V94 syn	6%
G09/Vero	1366	G	G>U	N406 syn	20.3%
	1481	G	G>U	N446 D>Y	18.5%
	2710	A	A>G	P245 Q>R	20.8%
	5638	U	U>A	F365 Y>N	38.7%
	6704	A	A>U	HN32 T>P	17.4%
	9896	G	G>U	L487 D>Y	45.4%

Interestingly the NGS revealed that a number of mutations were present at a low frequency (~3.9%) in the fusion related external domain (FRED) of the F protein in both passage 1 and passage 6 of PP1 and of MuV^G09^. These were already present in passage 1, which represents the 5^th^ passage in Vero cells after the original rescue and they were maintained at low frequency during the series. They affect the FRED domain by introduction of a number of charged residues that may well impact its functionality (Table 4). A similar observation was made in the NGS of the passage series of MuV^G09^ and thus this phenomenon is not specific to PP1 (see S3 Table for similar mutations in MuV^G09^).

**Table 4:** A cluster of mutations in the fusion related external domain at the N terminus of the F1 part of the fusion protein of PP1. N terminus of F1 103 FAGIA**I**G**I**AAALG**VATAAQV**T* 123.

Position	Mut	Effect	P1 geno	P1 anti	P6c geno	P6c anti
4869	U>G	F108 I>M	3/385	4/58	1/176	3/28
4873	A>U	F110 I>F	6/380	3/59	3/170	0/32
4881	A>C	F113 A syn	3/365	4/59	3/170	3/35
4884	C>G	F114 L syn	10/367	4/64	9/169	0/35
4888	G>U	F116 V>F	5/360	2/66	4/169	1/36
4892	C>A	F117 A>E	12/335	2/67	6/161	0/38
4894	A>C	F118 T>P	19/328	9/65	13/159	4/38
4898	C>A	F119 A>E	8/326	0/66	1/161	0/39
4901	C>A	F120 A>E	12/330	1/66	5/161	1/39
4903	C>A	F121 Q>K	22/329	7/66	11/159	1/38
4907	U>A	F122 V>E	20/327	1/69	11/156	2/42
total			120/3832	37/705	67/1811	14/400
frequency			3.1%	5.2%	3.7%	3.5%

* In large capitals the residues of the fusion related external domain that are affected by the mutations and in bold those that are changed as a result of a non-synonymous mutation.

In PP2 passage on B-LCL cells two variants seem to predominate with either a mutation at position 238 in the HN protein G > S or one at position 239 L > R. These are almost never present in the same RNA molecule and only a small number of original non-mutated reads remain. The significance of the two mutations observed in the HN gene of PP2 during the passage series in B-LCL cells cannot easily be assessed because this is a region of unknown significance.

Noticeable is also that in PP2 between positions 2227 and 2260 in the sixth passage about 6% of the reads showed a linked set of U to C mutations (Table 3) consistent with an interpretation that a number of biased hyper-mutated RNA molecules are carried along in passaged virus.

### The EGFP gene does not vary to a greater extent than the virus genes

*A priori* the expectation was that the EGFP gene in PP2 is not under selective constraint and that henceforth the ORF would have accumulated more mutations than the true virus genes. This appeared not to be the case when the number of variant readings at all positions in the ORFs encoding virus genes and that of EGFP were compared. The overall frequency of variant nucleotides at 0.20% was no greater in the EGFP ORF than in the other viral genes. The lack of selective constraint on this gene in contrast to those of the viral genes make this in our opinion (S4 Table) an unexpected result.

## Discussion

How and when during the rescue of PP1 and PP2 viruses the clusters of synonymous mutations arose is unclear. These replicating MuV were isolated as plaques from the wells in which the rescue experiment was performed. This is feasible because the rescue efficiency is relatively low and most wells in a six well plate do not contain more than one syncytium at 5–7 days post transfection. We have demonstrated here that once these clusters of mutations were generated the resulting virus populations were genetically stable when passaged 6 times with undiluted inoculum to allow for the maximum chance of the fixation of mutations. Deep sequencing of the virus RNAs did not show a bias towards reversion to the original nucleotide in the MuV^G09^ sequence. The direction of mutation appeared random and the variant readings at the original mutated positions in PP1 and PP2 were observed at such low frequencies that they are probably due to errors during the NGS sequencing procedure rather than representing true variants in the virus population. It is also clear that the stability of the PP1 and PP2 replicating viruses did not reflect an inability of the viruses to fix mutations during these passage series as variations did occur during the passages in other genes and nucleotide positions in both the PP1 and PP2 viruses as well as in the parent virus upon passage. These were often found at very high frequencies (3–45%) even though many were non-synonymous and probably reflect adaptation to the environment of a different host cell. The stability of the clustered mutations thus indicates that they contribute to a stable genotype that does not readily revert to the original MuV^G09^ set. Potential compensatory mutations in the V/P gene of PP1 and the N gene of PP2 were not observed as consistent features of the variations observed in NGS.

The stability of these mutant sets is remarkable. As they consist primarily of synonymous mutations or a small number of conservative amino acid replacements, it would be difficult to see a constraint at the protein coding level that would affect their reversion frequency. The maintenance of the set in repeat passaging may point to a higher order RNA structural constraint but these would be predicted to be operative only at the mRNA level as the RNA in the + and–strand RNPs appears devoid of secondary structure in the paramyxoviruses [17]. The positions of the mutant sets are interesting. They are located at the 3’ end of the genome. These areas have been shown to be preferentially sensitive to biased hyper-mutation caused by ADAR1 in measles virus [18]. Biased hyper-mutation (U to C) is also prevalent in these MuV samples in the N gene and the start of the V/P gene. The limited size of the sets does also not affect the overall codon usage in these replicating MuV, which is known to be a specific feature of each paramyxovirus [19] and the lack of synonymous mutations during viral evolution in the paramyxoviruses is observed but not explained.

How these mutant sets were generated in the first place is an open question and the most important one raised by the data. The phenomenon described here may be specific for rescue of MuV^G09^. In our experience with rescue of other paramyxo- and pneumoviruses such as measles, canine distemper, rinderpest virus and respiratory syncytial virus [20–23] as well as with some laboratory adapted strains of MuV- we have not encountered this phenomenon apart from occasional clusters of mutation that were generated by biased hypermutation involving primarily U to C and at a lesser frequency A to G changes. Since we have done this work with only a single clinical strain of MuV we are not able to link the phenomenon to the clinical or laboratory adapted nature of the virus. Furthermore, the rescue of infectious virus from plasmids in the system was not especially lees efficient than with the other viruses, which we have worked with. The clusters of mutations in the PP1 and PP2 viruses do not show this bias. The value for κ i.e. the ratio of transitions over transversions in all clusters summed together was 4.9 (bias towards transitions) which is similar to that found in between genotype comparisons for MuV. It is not due to the presence of the extra EGFP gene as it occurred both in PP1 and PP2.

It seems unlikely that the limited number of replications required to generate a syncytium during the primary isolation of these viruses would allow sequential selection of the large number of mutations that would provide these stable sets. Any explanation based on quasi-species theories would not only require a very large number of replications to generate the numbers of mutations but also a very strong positive selection for the second, third etc. synonymous mutation once the first had occurred. The observation that these mutations are clustered indicates a different mechanism for their generation. We suggest that the most likely processes that generated the clusters are either (i) the transcription of the DNA plasmid by T7 RNA polymerase or (ii) the early rounds of MuV replication by the RdRP operating in an environment different from normal virus infection into a host cell, followed by removal of unfit viruses by selection against viruses with lethal mutations in the N and V/P ORFs. Suggestions that they are generated by artefacts in the propagation of plasmids in *Escherichia coli* simply move the question as to how the clusters were generated one step back. The selection of a precise set of 11 in PP1 or 32 mutations in PP2 in *E*.*coli* that are synonymous and clustered in the virus genome and allow for the generation of replicating viruses is inexplicable and extremely unlikely. Furthermore, it should also be noted that most of the other plaque picked viruses did not contain these sets of clustered mutations but had the original MuV^G09^ sequence in the regions hyper-mutated in PP1 and PP2. Also recombination of the plasmid DNA with copies of the endogenized mumps virus sequences similar to the endogenous Borna virus like elements present in the genomes of several eukaryotes genomes [24] is unlikely as BLAST searches did not identify such sequences in the genome of the African Green monkey from which Vero cells are derived; similarly, no significant mumps virus N and V/P-like sequences were identified in the fowl pox genome.

Why the synonymous and conservative amino acid mutations occurred in clusters remains also an open question, but one possible explanation for the occurrence of the clusters of mutation has been suggested based on quantum biology. The genetic code is essentially a proton code in the plasmid DNA formed by the patterns of two (A-U) or three (G-C) hydrogen bonds mediated by protons that can tunnel between their alternative positions. Entanglement and tunnelling have been invoked in a number of studies dealing with mutations in biological systems [25, 26] and in chemistry are known as tautomerization. MacFadden and Al-Khahili [27] have speculated that a continuous process of quantum superposition and decoherence may allow a fast search for possible replicators from a dynamic combinatorial library. We suggest that in this case this may occur during the generation of primary transcripts from the full-length antigenomic DNA plasmid by T7 polymerase. On the basis of modelling quantum biological effects Rieper et al. [26] suggested that nucleotides in DNA might be read in the context of their neighbouring nucleotides, which may explain constraints on synonymous mutation in RNA viruses [19]. However, whilst quantum biological effects merit more attention in virology, their experimental verification as well as their counter-intuitive and speculative nature remain a challenge for biologists [27].

## Supporting information

S1 Table Titres of viruses obtained in the passage series of PP1 on Vero cells (A) and PP2 on B-LCL cells (B). (DOCX)

S2 Table Mutations observed in RNA extracted from the semi-purified PP1 virus after passage in Vero cells. (DOCX)

S3 Table Mutations observed in RNA extracted from the semi-purified MuVG09 after passage in Vero cells. (DOCX)

S4 Table Variations in the ORFS of MuV-PP2 after 6 passages in B-LCL cells. (DOCX)

## Abbreviations

PP1: rMuV^G09^PP1 plaque picked recombinant mumps virus
PP2: rMuV^G09^EGFP(3)PP2 plaque picked recombinant mumps virus expressing the EGFP gene between the V/P/I gene and the M gene
