# Supplementary material for: Unusual, stable replicating viruses generated from mumps virus cDNA clones
Source: PLoS One. 2019 Jul 5;14(7):e0219168. doi: 10.1371/journal.pone.0219168 (PMC6611571; doi:10.1371/journal.pone.0219168)
Supplement: S1 Table — (DOCX) [file pone.0219168.s001.docx]

**Table S1: Titres of viruses obtained in the passage series of PP1 on Vero cells (A) and PP2 on B-LCL cells (B).**

(A) Passages of MuV-G09 and rMuV-G09 PP1 on Vero cells.

| Passage | MuV-G09 | | | rMuV-G09- PP1 | | |
| --- | --- | --- | --- | --- | --- | --- |
|  | hpi | Titre | Comment | hpi | Titre | Comment |
| P0# | - | 4.2x10^4^ | 4 | - | 4.3x10^4^ | 2 |
| P1 | 50 | 7.5x10^4^ | 4 | 50 | 1.7x10^5^ | 2 |
| P2 | 47 | 1.3x10^5^ | 4 | 51 | 1.9x10^5^ | 2 |
| P3 | 47 | 2.0x10^5^ | 4 | 47 | 2.4x10^5^ | 2 |
|  |  |  |  |  |  |  |
| P3b | 50 | 2.0x10^5^ | 4 | 50 | 3.0x10^5^ | 2 |
| P4b | 48 | 7.6x10^4^ | 4 | 48 | 2.9x10^5^ | 4 |
| P5b | 80 | 7.0x10^3^ | 2 | 80 | 8.2x10^4^ | 4 |
| P6b | 72 | 1.5x10^6^ | 1,2,3 | 72 | 1.2x10^6^ | 1,3,4 |
|  |  |  |  |  |  |  |
| P3c | 58 | 2.4x10^6^ | 4,3 | 58 | 2.0x10^4^ | 2 |
| P4c | 48 | 4.0x10^6^ | 4 | 72 | 5.0x10^4^ | 2 |
| P5c | 48 | 1.9x10^6^ | 2 | 48 | 2.8x10^6^ | 4 |
| P6c | 47 | 5.0x10^5^ | 2 | 47 | 3.4x10^6^ | 4 |

# P0 is the fourth passage after rescue i.e. the first of which the sequence was determined. Two series of passages were made after passage 3 in order to determine whether they varied.

Comments

1: no cpe observed at harvesting

2: small plaques

3: interference prevented plaque formation at lower dilutions

4: normal size plaques

(B) Passages* of MuVG09(or) and rMuVG09EGFP PP2 on B-LCL cells

| Passage # | Time of harvest dpi | Titre G09 | Titre PP2 | Comments |
| --- | --- | --- | --- | --- |
| P1 | - | 4.6 x 10^3^ | 1.0 x 10^2^ | PP2 small plaques |
| P2 | 5 | 1.3 x 10^4^ | 3.0 x 10^2^ | PP2 small plaques |
| P3 | 5 | 1.3 x 10^5^ | 1.1 x 10^3^ | PP2 small plaques |
| P4 | 4 | 1.0 x 10^5^ | 3.0 x 10^3^ | PP2 normal plaques |
| P5 | 3 | 3.0 x 10^5^ | 1.2 x 10^5^ | PP2 normal plaques |
| P6 | 2 | 2.0 x 10^5^ | 2.0 x 10^5^ | PP2 normal plaques |

Titres determined by p.f.u./ml on Vero cells

*A passage is defined by removing 80% of the infected cell volume (6 ml) and adding 80% of uninfected cells (6 ml of fully grown B-LCL cells) back into the vessel.
