# Supplementary material for: Unusual, stable replicating viruses generated from mumps virus cDNA clones
Source: PLoS One. 2019 Jul 5;14(7):e0219168. doi: 10.1371/journal.pone.0219168 (PMC6611571; doi:10.1371/journal.pone.0219168)
Supplement: S2 Table — (DOCX) [file pone.0219168.s002.docx]

**Table S2 Mutations observed in RNA extracted from the semi-purified PP1 virus**

**after passage in Vero cells**

| Genome Position | Mutation | Passage 1 genomic | Passage 1 antigenomic | Passage 6b  genomic | Passage 6c  genomic | Passage 6c  antigenomic |
| --- | --- | --- | --- | --- | --- | --- |
| 14 | U>A |  |  | 2/3 |  |  |
| 849 | U>G |  |  |  | 5/90 |  |
| 1178 | G>A |  |  |  | 33/134 |  |
| 1547 | C>U | 42/291 | 11/115 |  |  |  |
| 1548 | C>U | 43/289 | 11/114 |  |  |  |
| 1563 | C>U | 39/274 | 10/118 |  |  |  |
| 1589 | C>U | 40/283 | 9/137 |  |  |  |
| 1590 | C>U | 40/283 | 9/137 |  |  |  |
| 1606 | C>U | 42/356 | 12/137 |  |  |  |
| 2583 | U>C |  |  |  |  |  |
| 2611 | A>C |  |  |  |  | 1/47 |
| 2634 | A>G |  |  |  |  |  |
| 2654 | A>G |  |  |  |  | 3/51 |
| 2807 | U>G |  |  |  |  |  |
| 3355 | A>G |  |  | 12/19 | 69/155 |  |
| 4176 | A>C |  |  |  | 2/131 |  |
| 4263 | G>A |  |  | 5/47 |  |  |
| 4857 | U>G |  |  |  |  | 2/27 |
| 5120 | U>G |  |  |  |  | 4/61 |
| 5863 | U>C |  |  |  |  | 4/70 |
| 8119 | G>A |  |  |  |  | 2/44 |
| 8378 | C>U | 4/375 |  |  |  |  |
| 9014 | U>C |  |  |  | 12/129 |  |
| 9364 | U>G |  |  |  |  | 2/30 |
| 9501 | U>G |  | 3/71 |  |  |  |
| 9608 | U>G |  |  |  |  | 2/52 |
| 10047 | U>G |  |  |  |  | 2/50 |
| 10071 | A>G |  |  |  |  | 2/44 |
| 11425 | U>C |  | 3/55 |  |  |  |
| 11720 | C>U |  |  |  |  | 2/31 |
| 12748 | C>U |  |  |  | 44/217 |  |
| 14490 | U>C |  |  |  |  | 9/510 |
| 14504 | U>C |  |  |  |  | 15/521 |
| 14587 | U>C |  |  |  |  | 13/426 |
| 14758 | A>C |  |  |  |  | 9/571 |
| 15225 | U>C | 101/5209 |  |  |  | 26/735 |

Empty cells indicate that no variant reads were observed.
