# Supplementary material for: Unusual, stable replicating viruses generated from mumps virus cDNA clones
Source: PLoS One. 2019 Jul 5;14(7):e0219168. doi: 10.1371/journal.pone.0219168 (PMC6611571; doi:10.1371/journal.pone.0219168)
Supplement: S3 Table — (DOCX) [file pone.0219168.s003.docx]

**Table S3 Mutations observed in RNA extracted from the semi-purified MuVG09**

**after passage in Vero cells**

| GenomePosition | Mutation | Passage 1 genomic | Passage 1 antigenomic | Passage 6b  genomic | Passage 6b  antigenomic | Passage 6c  genomic | Passage 6c  antigenomic |
| --- | --- | --- | --- | --- | --- | --- | --- |
| 14 | U>A | 3/31 | 21/80 |  |  |  | 73/183 |
| 1366 | G>U |  |  |  |  | 249/1174 | 64/369 |
| 1481 | G>U |  |  |  |  | 165/946 | 76/354 |
| 1547 | U>C |  |  |  |  |  | 77/408 |
| 1562 | U>C |  |  |  |  | 76/844 | 68/380 |
| 1572 | U>C | 2/138 |  |  |  |  |  |
| 1575 | U>C |  | 2/243 |  |  | 56/796 | 72/395 |
| 1589 | U>C |  | 3/240 |  |  | 60/743 | 68/380 |
| 1598 | U>C | 1/108 | 2/240 |  |  | 89/785 | 58/373 |
| 1645 | U>C |  |  |  |  | 180/947 | 66/388 |
| 2710 | A>G |  |  |  |  | 114/564 | 77/356 |
| 4816 | G>A |  |  |  |  | 178/923 | 55/215 |
| 4884 | C>G | 16/332 | 2/89 |  |  | 42/935 |  |
| 4890 | U>G | 5/315 | 3/92 |  |  |  |  |
| 4894 | A>C | 13/323 | 18/96 |  |  |  | 25/153 |
| 4898 | C>A | 11/334 | 1/96 |  |  |  |  |
| 4903 | C>A | 25/335 | 6/99 |  |  |  | 15/156 |
| 4907 | U>A | 9/324 | 3/104 |  |  |  |  |
| 5638 | U>A |  |  |  |  | 438/1065 | 55/210 |
| 5793 | C>A |  |  |  |  |  | 48/266 |
| 6699 | U>C | 46/378 | 8/83 |  |  |  |  |
| 6704 | A>C |  |  |  |  |  | 37/212 |
| 7599 | A>G |  |  |  |  |  |  |
| 8243 | A>U | 6/723 |  |  |  |  |  |
| 8453 | G>U |  |  |  |  |  | 6/165 |
| 8459 | G>A |  |  |  |  |  | 13/170 |
| 8482 | C>A |  |  |  |  |  | 10/175 |
| 9896 | G>U |  |  |  |  | 487/1033 | 53/157 |
| 10180 | G>U | 450/548 | 99/116 |  |  | 370/1584 | 93/239 |
| 10946 | A>G |  |  |  |  | 331/754 | 63/151 |
| 11534 | A>G |  | 6/65 |  |  |  |  |
| 12545 | A>G |  |  |  |  | 56/1710 | 5/206 |
| 13716 | U>C |  | 12/349 |  |  |  |  |
| 13768 | U>C |  | 13/380 |  |  |  |  |
| 13778 | A>G |  | 2/390 |  |  |  |  |
| 13785 | U>C |  | 5/273 |  |  |  |  |
| 13801 | U>C |  | 4/282 |  |  |  |  |
| 13810 | U>C |  | 4/275 |  |  |  |  |
| 14870 | A>G |  |  | 31/796 | 25/846 |  |  |
| 14882 | A>G |  |  | 38/849 |  |  |  |
| 14908 | A>G |  |  | 24/545 |  |  |  |
| 14909 | A>G |  |  | 26/550 | 20/755 |  |  |
| 14923 | A>G |  |  | 8/571 |  |  |  |
| 15049 | A>G |  |  |  |  |  |  |
| 15052 | G>U |  |  | 15/217 |  |  |  |
| 15054 | G>U |  |  | 17/230 |  |  |  |
| 15285 | U>G | 419/4288 | 182/1739 | 179/237 | 238/337 | 3382/6054 | 2560/3527 |
| 15354 | Insert A |  |  |  |  |  |  |

Empty cells indicate that no variant reads were observed.

Blocks of positions coloured red/blue indicate linked mutations present on the same reads
