# Supplementary material for: Unusual, stable replicating viruses generated from mumps virus cDNA clones
Source: PLoS One. 2019 Jul 5;14(7):e0219168. doi: 10.1371/journal.pone.0219168 (PMC6611571; doi:10.1371/journal.pone.0219168)
Supplement: S4 Table — (DOCX) [file pone.0219168.s004.docx]

**Table S4 Variations in the ORFS of MuV-PP2 after 6 passages in B-LCL cells.**

| ORF | Overall  Variation |
| --- | --- |
| N | 0.18% |
| P | 0.25% |
| EGFP | 0.24% |
| M | 0.22% |
| F | 0.21% |
| SH | 0.26% |
| HN | 0.30% |
| L | 0.32% |
